# Heart rhythm assessment in elite endurance athletes: A better method?

**DOI:** 10.3389/fspor.2022.937525

**Published:** 2022-07-25

**Authors:** Ådne Ausland, Edvard Liljedahl Sandberg, Jarle Jortveit, Stephen Seiler

**Affiliations:** ^1^Department of Sport Science and Physical Education, Faculty of Health and Sport Sciences, University of Agder, Kristiansand, Norway; ^2^Department of Cardiology, Sorlandet Hospital, Arendal, Norway

**Keywords:** atrial fibrillation, endurance athletes, elite athletes, cardiac screening, cardiac arrhythmia

## Abstract

**Introduction:**

Arrhythmias also occur among elite endurance athletes. Conventional diagnostic tools for assessment of arrhythmias suffer from limited availability and usability challenges, particularly under the demanding training conditions of an elite athlete. Among endurance athletes, there is a need for out-of-hospital monitoring to enhance detection of arrhythmias under conditions that are relevant and potentially provocative of underlying pathology. The Norwegian patch ECG247 Smart Heart Sensor has been developed to simplify the assessment of heart rhythm disorders. The current study aimed to evaluate the ECG247 Smart Heart Sensor function and usability in an elite athlete environment.

**Methods:**

A total of 13 professional cyclists from the UNO-X Pro Cycling Team were examined with the ECG247 Smart Heart Sensor during training camp in Spain, December 2021. All ECG data were analyzed by cardiologists at Sorlandet Hospital Arendal, Norway. The athletes also completed a brief questionnaire registering their training (from on-bike monitoring units) and provided self-assessment of usability parameters after the test.

**Results:**

In 8 of 13 athletes (69% male, age 23 ± 4 years), two test periods were performed with different ECG patches, resulting in a total of 21 tests with continuous ECG monitoring. Average total ECG test duration per athlete was 144 ± 47 h (89 ± 24 h/patch). Athletes performed an average of 15 ± 5 training h during each test. The ECG quality from all tests was considered satisfactory for rhythm analysis—also during exercise. The reported usability of the ECG247 Smart Heart Sensor was high, and no athletes reported trouble sleeping or training with the sensor. The automatic arrhythmia algorithm reported episodes of possible arrhythmias in 5 (24%) tests; 2 atrial flutter, 2 supraventricular tachycardia and 1 bradycardia (heart rate <30/min). Manual assessment by physicians verified the episode of bradycardia but revealed normal sinus rhythm in all other tests. No false negative events were identified in over 1,800 h of ECG collection.

**Conclusion:**

The ECG247 Smart Heart Sensor allowed for high quality ECG monitoring with high usability during intensive exercise in athletes.

## Introduction

The importance of large volumes of training to perform at a high level in endurance sports is well documented among elite athletes (Seiler, 2010; Tønnessen et al., 2014; Stöggl and Sperlich, 2015). Elite endurance athletes' annual training volume typically ranges from 500 to well above 1,000 h (Billat et al., 2001; Tønnessen et al., 2014; Metcalfe et al., 2017). Endurance exercise is also established as an efficacious method of reducing the risk of developing cardiovascular diseases (CVD). However, there are multiple studies suggesting that “excessive” long-lasting and high-volume endurance training may paradoxically increase the risk of developing certain types of heart disease, particularly arrhythmias (Madias, 2008; Goodman et al., 2015). Atrial Fibrillation (AF) is one of the most common cardiac arrhythmias reported among endurance athletes and AF incidence in athletes has been a theme of considerable research interest (Grimsmo et al., 2011; Andersen et al., 2013; Sanchis-Gomar et al., 2016; Lippi et al., 2021; Newman et al., 2021).

Today's gold-standard for diagnosing cardiac arrhythmias is a 12-lead electrocardiogram (ECG). An ECG test is performed by healthcare personnel in a clinical setting and provides a time-limited snapshot of the heart's electrical function (Quer et al., 2020). Some specific cardiac arrhythmias may be transient, such as AF, and a 12-lead ECG recording period lasting only a few minutes may fail to detect intermittent cardiac arrhythmias. Continuous ECG-recordings are needed to detect and diagnose specific cardiac arrhythmias and the equipment used for long-term ECG recordings is often referred to as “Holter monitoring” (Kułach et al., 2020). A Holter monitor system typically requires a recording device worn on the hip and coupled to at least three cables attached to electrodes on the chest. The system is applied to the patient by specialized healthcare professionals, and is usually worn for ~24–72 h (Lutfullin et al., 2013). Most Holter systems are not water repellent. Consequently, the Holter monitor system may limit movements and can loosen or detach with physical activity and hard exercise.

For an elite athlete training daily, a Holter monitor prescription will prevent the athlete from training normally, thereby decreasing the validity of the ECG monitoring process. Arrhythmias among elite athletes often occur during exercise (Madias, 2008). A Holter monitor may limit the intensity or continuity of the exercise (Lutfullin et al., 2013). 12-lead ECG and Holter monitoring are dependent on assistance from healthcare personnel, and therefore are subject to limited availability, limited test duration time, and usability challenges. In the context of a high-performance endurance sport team, cardiac screening with today's clinical tools becomes so time consuming that it may be avoided by athletes and coaches despite the appearance of symptoms of concern.

Several new systems purporting to provide long-term ECG monitoring are available. “Smart” watches and training accessories can provide identification of arterial pressure waves. However, international guidelines require ECG documentation for the diagnosis of arrhythmias. Self-applied, single lead ECG patches are currently available on the market for home-based use. However, there is a lack of research evidence regarding their validity and utility in a high-performance endurance athlete population. TheECG247™ Smart Heart Sensor is a new, mobile, long-term patch ECG monitoring device that has undergone extensive testing in a home health care setting (Sandberg et al., 2021; Jortveit and Fensli, 2022; Jortveit et al., 2022) and is approved by European directives for medical devices (93/42/EEC). It provides continuous monitoring of the heart rhythm for up to 7 days and can be used during exercise. The device is small, wireless, and easy to apply and use without any clinical expertise or assistance (Appsens, 2021). All the data acquired by the sensor is uploaded to cloud storage through a smartphone application and can be easily accessed by health care professionals. The user also has access to real-time ECG feedback during testing. The ECG sensor patch is applied over the sternum and remains attached through the whole monitoring period. Monitoring duration is limited by the integrity of the fixation of the sensor patch to the skin over time (up to 7 days). ECG247 has not been systematically tested on athletes. If this ECG patch technology withstands the use characteristics of elite athletes (vigorous movement, sweat, showers, etc.), it can potentially become a viable alternative for screening and cardiac rhythm monitoring in athletes.

The aims of this study were: (1) to evaluate how the ECG247 Smart Heart Sensor technical solution performs in a setting representative of the demands of high-performance endurance athletes during daily training, (2) to investigate the perception of comfort and usability among elite athletes training in demanding field conditions, and (3) to evaluate the ECG quality and automatic arrhythmia detection during high endurance training.

## Materials and methods

### Study design

This study was designed as a descriptive field test of the ECG247 Smart Heart Sensor (Appsens AS, Lillesand, Norway) on elite endurance athletes from the Uno-X Pro Cycling Team while performing a high volume of endurance training. Methods were designed to accommodate the practical demands of the athletes while assessing both technical and practical aspects of using the ECG device in a sports medicine context. All data collection and testing were performed in December 2021.

### Study subjects and procedure

The field test was completed during a 14-day training camp for the Uno-X Pro Cycling Team in Spain, December 2021, and a total of 13 athletes (9 male) were monitored with the ECG247 sensor. These athletes were selected from the entire team (~50 athletes) by the Uno-X team leadership. They participated in an information meeting and provided signed informed consent prior to the start of ECG data collection. The athletes agreed to wear the sensor for 3–6 days (depending on quality of the ECG recording). The research project leader was present at the training camp during the test period and answered questions from athletes. During the training camp, collected ECG recordings were simultaneously reviewed by physicians at Sorlandet Hospital Arendal in Norway. Cardiological support was provided during the field-testing period to ensure rapid communication with athletes in the event of detected arrhythmias or if false positive events arose. After completion of the field-testing period, the physicians performed a manual review of the complete ECG recordings from every athlete and provided a detailed report for each athlete. Acceptable ECG quality was defined as the ability to determine rhythm (sinus rhythm or specific arrhythmia) based on the physician's assessment. In case of doubt, additional physicians (cardiologists) were consulted.

### ECG247 smart heart sensor

**Figure 1:**
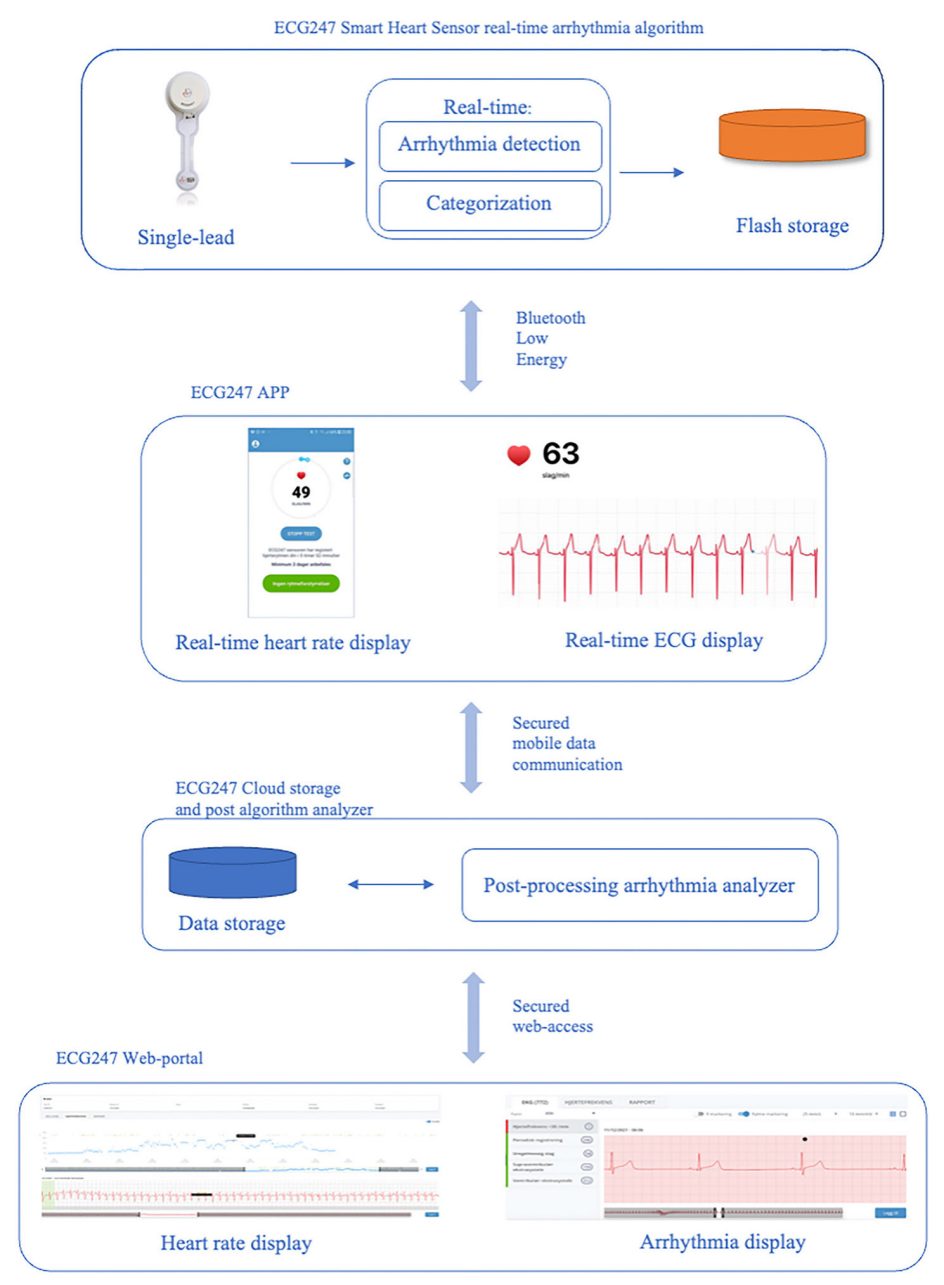
The ECG Smart Heart Sensor system: sensor with real-time arrhythmia detection, smartphone application, back-end cloud service with post-processing arrhythmia analyzer, and web portal.

**Figure 2:**
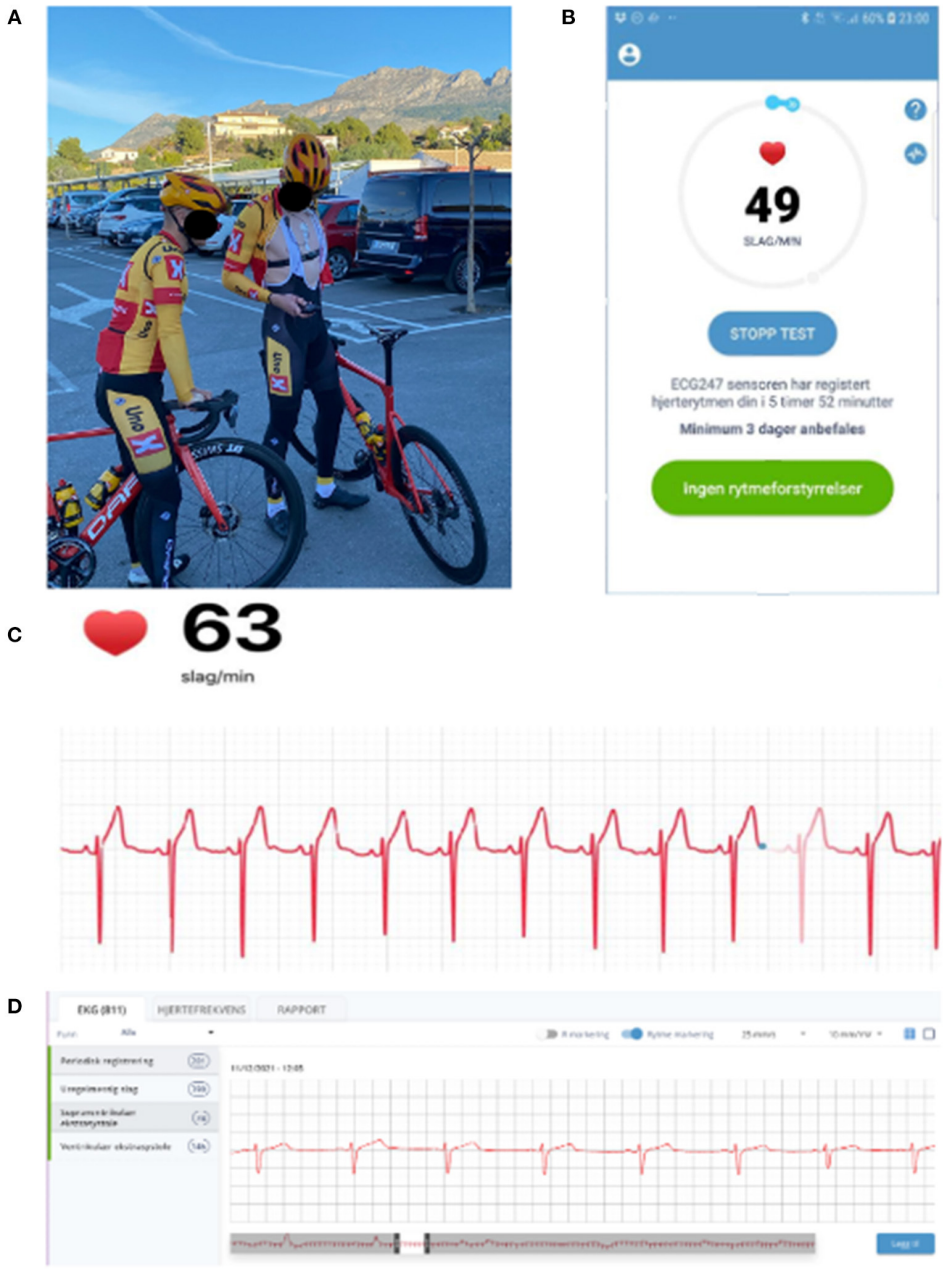
**(A)** The ECG247 sensor placed over the sternum, screenshots from **(B,C)** the ECG247 mobile application and **(D)** the web portal.

The ECG247 is a single-lead patch ECG-monitoring device. The monitoring system consists of a one-time “multi-day use” electrode patch that is attached over the sternum, a re-usable ECG sensor, a smartphone application, a back-end cloud service, and a web portal (Figure 1). The ECG247 sensor continuously monitors the hearth rhythm and automatically detects and categorizes arrhythmias in real-time by using algorithms based on artificial intelligence in the sensor and at the back-end service. The ECG247 Smart Heart Sensor system and the arrhythmia detection algorithms are described previously (Sandberg et al., 2021; Jortveit and Fensli, 2022). The ECG-recordings are transferred *via* Bluetooth to the ECG247 application on the smartphone, and simultaneously uploaded to the back-end cloud service (Figure 2). In cases when the Bluetooth communication between the sensor and the smartphone is interrupted, the ECG247 application will send a notice, with reestablishment of the connection made automatically. In addition, the sensor has an internal memory for ECG storage in case communication error with the phone. The user has ownership and access to the results in the web portal and can provide permissions for sharing of ECG data with their physician or other healthcare professionals. User authentication is provided using the Firebase Service (Google, Mountain View, CA, USA), which generates a two-factor authentication required for access to sensitive health information. All information stored in the web portal is coded as Fast Health Interoperability Resources (FHIR).

All detected arrhythmias are uploaded and saved in the back-end cloud service and sorted by severity in the web portal. The user can also manually highlight up to 1 min of ECG recording by activating this function on the sensor. This allows the user to “tag” ECG measurements when they subjectively experience what they perceive to be a disturbance in heart rhythm.

### Laboratory ECG smart heart sensor pilot test

**Figure 3:**
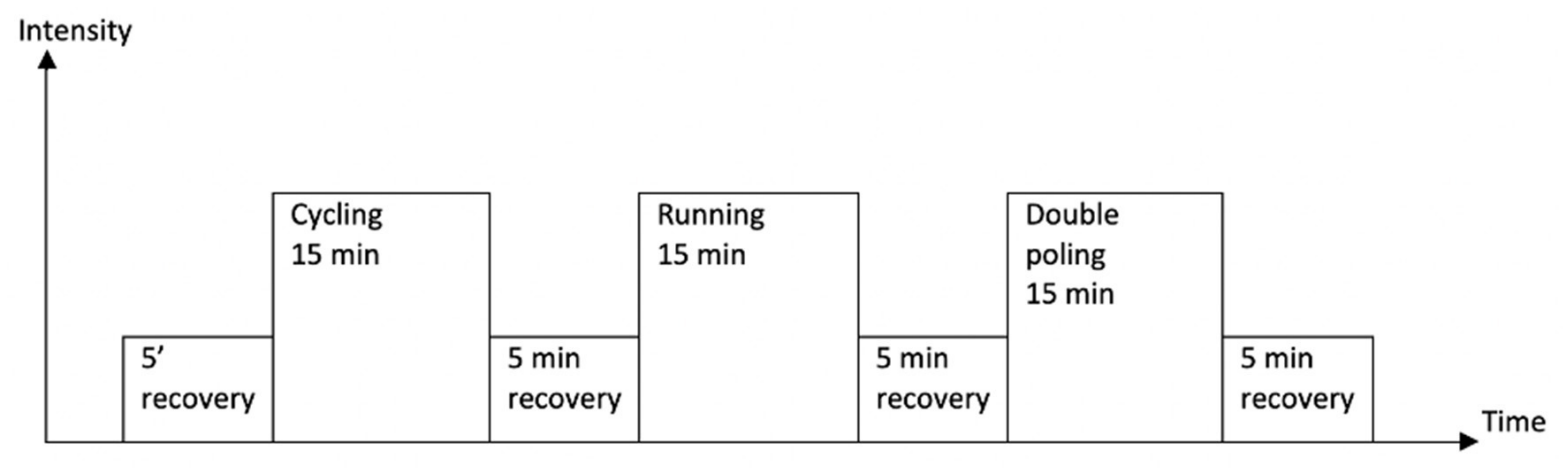
Test protocol for the pilot test of the ECG247. Started with applying the sensor and connect to the participants phones. Recovery consisted of walking and sitting. The intensity increased slightly every 5 min during the efforts.

**Figure 4:**
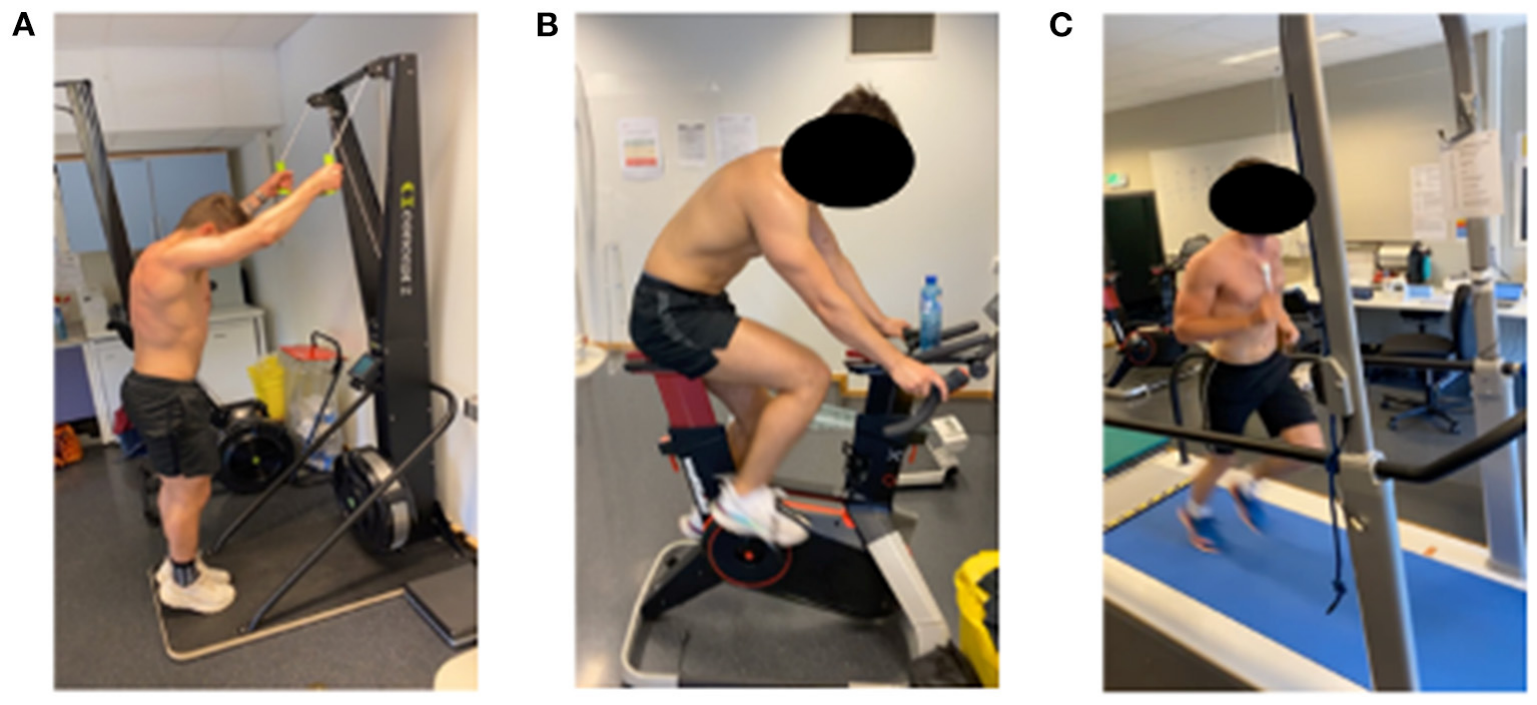
Laboratory exercise modalities evaluated during preliminary testing of ECG24 sensor: **(A)** Ski double-poling, **(B)** Cycling, **(C)** Running.

Prior to the field testing of ECG247, preliminary pilot testing in the laboratory was conducted on 6 (4 male, 2 female) physically active sport science students. The primary purpose of the pilot test was to investigate how different movements (cycling, double-poling, running) affected the ECG recordings, as well as evaluate the tolerance of the single-use electrode for repeated bouts of exercise and showering. The positive results of this preliminary test were also deemed a necessary pre-condition for further testing with UNO-X Pro Cycling Team. The test protocol in the laboratory consisted of 15 min efforts on each exercise modality. These efforts were divided into 5 min segments with small successive increases in work intensity. A 5 min rest period was provided between each 15 min exercise bout (Figure 3). Double-poling (Figure 4A) was performed on a Concept2 Skierg (Concept2, Morrisville, VT, USA), cycling (Figure 4B) on a Wattbike AtomX (Wattbike, Nottingham, England), and running (Figure 4C) on a motorized treadmill (Lode Katana Sport, Lode B. V., Groningen, Netherlands).

### Ethical considerations

The study was carried out according to the Declaration of Helsinki and data collection methods were approved from a data security perspective by the Norwegian Center for Research Data and was approved by the Ethics Committee of the Faculty for Health and Sport Science, University of Agder.

Athlete participants were not randomly selected by Uno-X team leadership. Athletes with history of reporting possible arrhythmic symptoms were selected to be among the test participants to participate in the test. Consequently, a cardiologist was brought in early to provide additional information to the athletes. In this process, the cardiologist informed the participants that the current algorithms of the ECG247 were not specifically designed for athletes exercising at high heart rates. This increased the likelihood of false positive detection of supraventricular tachycardia (SVT) and Atrial Flutter (AFLU) when heart rate (HR) was normally elevated during training sessions. Therefore, false positive events related to these tachycardias were anticipated and discussed with the athletes.

## Results

### Laboratory ECG247 smart heart sensor pilot test

**Figure 5:**
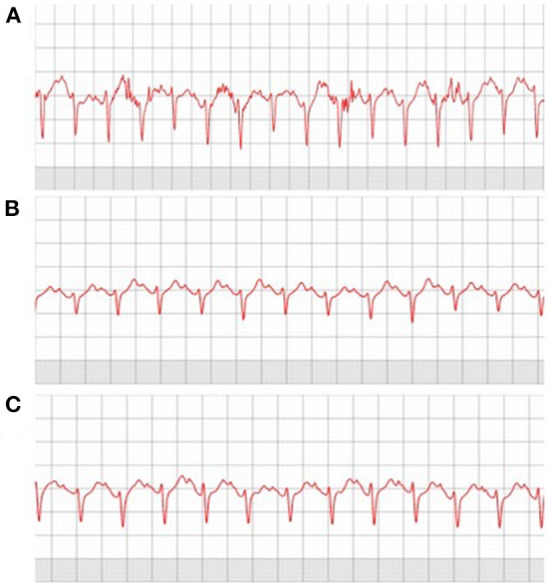
Laboratory exercise modalities evaluated during preliminary testing of ECG247 sensor: **(A)** Running, **(B)** Cycling, **(C)** Double poling XC.

A total of 6 (4 male) subjects completed the preliminary pilot testing in the laboratory. Figure 5 demonstrates the ECG recordings of the different modalities of one of the subjects. Running (Figure 5A) showed more disturbance in the ECG recordings among all subjects compared with cycling (Figure 5B) and XC-ski double-poling (Figure 5C). All ECG recordings were evaluated by a cooperating physician within 48 h of the test period and considered satisfactory for rhythm analysis in all the tests.

### Field test of the ECG247 smart heart sensor

Continuous ECG monitoring was successfully performed on a total of 13 athletes. The average age of the participants was 23 ± 4 years (69% males). In 8 of 13 athletes, 2 test periods were performed, resulting in 21 continuous ECG monitoring periods of at least 43 h. New tests, with new single use electrode patches were started due to partial detachment of the electrode from the skin (*n* = 1), ECG signal degradation was identified remotely by the physician (*n* = 4), and by request from athletes (*n* = 3). The mean athlete test duration time was 144 ± 47 h, with an average functional duration of 89 ± 24 h for each ECG patch/test period. During the test period, an average of 24 ± 6 h of training was performed by each athlete, with an averaging 15 ± 5 training h for each electrode patch.

### Self-reported usability of ECG247 smart heart sensor

Four participants reported some discomfort (itching) underneath the sensor patch on the chest. Three of these four athletes reported that the itching stopped after the first 24 h of the test. Nine athletes reported forgetting that they were wearing the sensor from time to time. No athletes reported trouble sleeping or training with the ECG247 sensor. None of the 13 tested athletes reported problems with the connection or Smart phone application. However, 7 athletes reported concerns and questions around results during the test period (Table 1).

**Table 1:** Usability of ECG247 smart heart sensor.

	**All (*****n*** = **13)**
Itching	4
No reported discomforts	9
Disturbed sleep	0
Disturbed training	0
Disturbed phone connection	0
Concerns during the test	7

*Values are presented as prevalence*.

*n, number of participants*.

### ECG quality and automatic arrhythmia detection

The ECG quality from all tests was considered satisfactory by the physicians for rhythm analysis—also during exercise. One short nocturnal episode of bradycardia (heart rate <30/min) was detected by the ECG247 automatic algorithms and verified by the physician. Two short episodes of SVT and 2 short episodes of AFLU in four different athletes were marked by the ECG247 system, but all of these were refuted by the manual assessment of the physicians. User-initiated recordings were performed five times without any pathological ECG findings (Table 2).

**Table 2:** Characteristics and diagnostic evaluation for the field tests.

	**Athletes** **(*****n*** = **13)**	**Tests** **(*****n*** = **21)**
Age (y)	23 ± 4	
Test duration (hours)	144 ± 47	89 ± 24
Training volume (hours)	24 ± 6	15 ± 5
Showers (times)	6 ± 1	4 ± 1
Recording periods <72 h	3	3
ECG247 algorithm detection
AF and severe arrhythmia	0	0
Bradycardia	1	1
False positive SVT	2	2
False positive AF	2	2
False negative	0	0
Patient-initiated recordings
Recordings	3	5
Physician review detection of arrythmia	1	1

*Values are presented as mean ± standard deviation and prevalence*.

*SVT, supraventricular tachycardia; AFlu, atrial flutter; n, number of participants*.

## Discussion

This study of the ECG247 Smart Heart Sensor technical performance in 13 endurance athletes from the Uno-X Pro Cycling Team during extensive training verified technical quality, usability, and ECG quality satisfactory for heart rhythm assessment, also during exercise.

The findings of the present study suggest that the ECG247 Smart Heart Sensor provides an easy and technical acceptable method of monitoring cardiac health in athletes with minor negative side effects or annoyances. The system overcomes the limitations of conventional diagnostic tools for assessment of rhythm disorders like limited availability, limited test duration time, and usability challenges, particularly under the demanding training conditions of an elite athlete.

The reported usability of the ECG247 Smart Heart Sensor was high, and no athletes reported trouble sleeping or training while wearing the sensor. The project leader present at the training camp during the field testing received athlete concerns during the test period. These concerns arose mainly from reports from the application saying that there was a possible arrhythmia. Most of these events were determined to be false positive. The patch sensor showed promising usability also in a team training camp context. The sensor enables transition of the assessment of arrhythmias from the hospitals to the athlete's training and competition environment. Professional sports teams are often composed of multinational athletes, with different healthcare service providers. An out-of-hospital, reusable cardiac rhythm device could make assessment of heart rhythm disorders and heart symptoms cheaper and less time-consuming compared with conventional hospital methods. In addition, ECG247 Smart Heart Sensor did not limit exercise in any way, which is a crucial detail when monitoring elite athletes.

A purpose of the pilot test was to investigate how different movements (cycling, double-poling, running) affected the ECG recordings, as well as initially evaluate the tolerance of the single-use electrode for repeated bouts of exercise and showering. The pilot testing completed as a prelude to the present study illustrates that there are some differences in ECG quality across exercise modalities. There was one incident of a false positive test (AFLU) during preliminary lab testing, which provided perspectives about the need to ensure the safety and psychological wellbeing of the athletes during the field trial. A cardiologist was brought in to analyze potential arrhythmias simultaneously during the test period. The quality of the ECG recordings was considered satisfactory for hearth rhythm assessment in the pilot test. However, more work is needed on the different exercise modalities and their potential influence on the quality of the ECG recordings.

The findings from the present field testing will inform algorithm adaptation for sport medicine applications. This athlete population represented a severe test of the technical solution given the high training volumes performed. The capacity of the solution to deliver continuous, interpretable ECG recordings for at least 48 h was deemed as a cutoff for minimum viability in a sports medicine context. The arrythmia detection algorithms employed were originally based on a sedentary, primarily elderly population. SVTs and AFLU may be near identically to the ECG of an athlete exercising with abrupt changes in HR.

Therefore, the physician on the research team anticipated a risk of false positive findings associated with the high heart rates achieved during normal training in this elite athlete group. Prior to the field test, athlete volunteers were informed that the integrated arrhythmia analyzing algorithm was sensitive to abrupt HR elevation and might falsely detect events of AFLU and SVTs However, the proportion of false positive arrhythmia events detected by the automatic algorithm was relatively low (19%). Importantly, no actual ECG arrythmias went undetected (false negative) by the algorithmic solution in over 1,800 h of ECG monitoring.

### Strength and limitations

The main strengths of the present study were: (a) cooperation with a professional cycling team, which provided an excellent field-testing environment, and (b) extensive preliminary pilot testing. A training camp, with a professional cycling team was an appropriate environment for testing whether ECG247 Smart Heart Sensor withstands the typical patterns of athlete training several hours daily, showering, etc. In addition, testing the sensor during a training camp was a good simulation for investigating how it works in a team context. On-time access to a cardiologist was crucial for this initial study because it provided both reassurance for the athletes and ensured an optimal analysis process. However, this was not an interventional study, and there was no comparison with today's best practice (Holter monitoring). Cycling is also one of the endurance sports with the least amount of movement in the upper body. Therefore, the present findings should not be generalized to all sports movements.

A single-lead ECG may be more difficult to interpret by a physician compared to a 3-lead ECG from a Holter system. However, the number of leads is less important for the interpretation of narrow QRS complex arrhythmias like AF and SVT. The position of the single-lead ECG patch is essential for high signal quality on the ECG recordings during physical exercise. The ECG247 Smart Heart Sensor is placed directly over the sternum. This is an anatomical placement with little multi-directional skin stretch and muscular movement under the electrode and therefore presumably less electrical signal disturbance compared with placing the sensor left on the chest, over skin and muscle that introduces significant resonant movement artifact during exercise. For sport-use, sternal ECG electrode placement of single-lead patch electrodes may be optimal.

### Practical applications

The importance of large volumes of training to perform at a high level in endurance sports is well documented among elite athletes (Seiler, 2010; Tønnessen et al., 2014; Stöggl and Sperlich, 2015). These findings highlight the need for cardiac screening methods which are easily accessible and do not interfere with the everyday training of an elite athlete. The field test of ECG247 Smart Heart Sensor illustrates how assessment of possible heart rhythm disorders can be performed in an elite team environment, without any interference of training and sleeping rhythms.

As mentioned, cycling is one of the endurance sports with the least amount of movement in the upper body. Therefore, additional field testing of this device in other athlete groups, such as runners, is warranted.

## Conclusion

The study demonstrates that the ECG247 Smart Heart Sensor allowed high quality ECG monitoring with high usability during intensive exercise in athletes.

## Data availability statement

The raw data supporting the conclusions of this article will be made available by the authors, without undue reservation.

## Ethics statement

The studies involving human participants were reviewed and approved by Ethics Committee of the Faculty for Health and Sport Science, University of Agder. The patients/participants provided their written informed consent to participate in this study.

## Author contributions

ÅA and SS was responsible for the data collection. ES was responsible for manual assessment of the ECG data. ÅA drafted and SS, ES, and JJ critically revised the manuscript. All authors gave final approval and agreed to be accountable for all aspects of this work.

## Conflict of interest

ES has received speaking fees from Pfizer. JJ has received speaking fees from Amgen, AstraZeneca, BMS, Boehringer Ingelheim, Novartis, Pfizer, and Sanofi. He is a shareholder in AppSens AS and is employed in the company. The remaining authors declare that the research was conducted in the absence of any commercial or financial relationships that could be construed as a potential conflict of interest.

## Publisher's note

All claims expressed in this article are solely those of the authors and do not necessarily represent those of their affiliated organizations, or those of the publisher, the editors and the reviewers. Any product that may be evaluated in this article, or claim that may be made by its manufacturer, is not guaranteed or endorsed by the publisher.
